# Characteristics of HIV patients who missed their scheduled appointments

**DOI:** 10.1590/S0034-8910.2015049005145

**Published:** 2016-01-12

**Authors:** Delsa Nagata, Eliana Battaggia Gutierrez

**Affiliations:** I Programa de Pós-Graduação em Doenças Infecciosas e Parasitárias. Faculdade de Medicina. Universidade de São Paulo. São Paulo, SP, Brasil; II Divisão de Clínica de Moléstias Infecciosas e Parasitárias. Instituto Central do Hospital das Clínicas. Faculdade de Medicina. Universidade de São Paulo. São Paulo, SP, Brasil

**Keywords:** Acquired Immunodeficiency Syndrome, HAART, Medication Adherence, Continuity of Patient Care, Appointments and Schedules, Health Information Systems

## Abstract

**OBJECTIVE:**

To analyze whether sociodemographic characteristics, consultations and care in special services are associated with scheduled infectious diseases appointments missed by people living with HIV.

**METHODS:**

This cross-sectional and analytical study included 3,075 people living with HIV who had at least one scheduled appointment with an infectologist at a specialized health unit in 2007. A secondary data base from the Hospital Management & Information System was used. The outcome variable was missing a scheduled medical appointment. The independent variables were sex, age, appointments in specialized and available disciplines, hospitalizations at the Central Institute of the Clinical Hospital at the Faculdade de Medicina of the Universidade de São Paulo, antiretroviral treatment and change of infectologist. Crude and multiple association analysis were performed among the variables, with a statistical significance of p ≤ 0.05.

**RESULTS:**

More than a third (38.9%) of the patients missed at least one of their scheduled infectious diseases appointments; 70.0% of the patients were male. The rate of missed appointments was 13.9%, albeit with no observed association between sex and absences. Age was inversely associated to missed appointment. Not undertaking anti-retroviral treatment, having unscheduled infectious diseases consultations or social services care and being hospitalized at the Central Institute were directly associated to missed appointments.

**CONCLUSIONS:**

The Hospital Management & Information System proved to be a useful tool for developing indicators related to the quality of health care of people living with HIV. Other informational systems, which are often developed for administrative purposes, can also be useful for local and regional management and for evaluating the quality of care provided for patients living with HIV.

## INTRODUCTION

In terms of universal access to highly active antiretroviral therapy (HAART), one challenge to achieving greater effectiveness and impact from the therapy for people living with HIV (PLHIV) is maintaining high HAART adherence rates.^8^ ^,^ ^18^ HAART success was associated with consultation attendance rates.^1^ ^,^ ^18^ In addition, the number of missed consultations was associated with a risk of death,^17^ ^,^ ^20^ ^,^ ^23^ and the total days lost between missing an appointment and the following one was associated with new AIDS-defining illnesses.^20^

Sex,^12^ age,^3^ ^,^ ^11^ ^,^ ^12^ ^,^ ^17^ ethnicity,^3^ ^,^ ^12^ ^,^ ^17^ low CD4 count,^17^ using alcohol and drugs,^17^ not using antiretroviral drugs,^11^ little social^3^ ^,^ ^17^ or family support^1^ and loss of social security^17^ were identified as factors associated with missing medical consultations/appointments.

From a management point of view, scheduled consultation absences are a problem for both health services and society, these aspects can be translated into financial costs.^2^ ^,^ ^22^ How the health service, directed towards PLHIV care, is organized is an important aspect related to HAART adherence.^19^ Barriers to service access and to information, lack of regularity in attending scheduled consultations and in collecting medications can be negatively associated to HAART adherence.^8^

This study aimed to analyze whether sociodemographic characteristics, consultations and care in specialized services are associated with scheduled infectiological consultations missed by people living with HIV.

## METHODS

This study is retrospective, analytical cross-sectional in nature and was developed within the HIV/AIDS Patient Care Extension Service (SEAP HIV/AIDS) at the Clinical Division of Infectious and Parasitic Diseases attached to the Central Institute of the Clinical Hospital at the Faculdade de Medicina da Universidade de São Paulo (ICHCFMUSP), which is an outpatient and day hospital service specialized in providing services to people living with HIV. The study period from which all data were taken was 2007. During this period, the unit dealt with approximately 3,000 HIV/AIDS adults through the Brazilian Unified Health System (SUS). The consultations were scheduled by appointment; unscheduled consultations were also available to meet spontaneous demand.

The information included in this study was obtained from a Hospital Management & Information System (SIGH), which is a hospital administrative system, developed by the Data Processing Company of the State of Sao Paulo (PRODESP), for production and billing. The system is widely used by SUS State health services, including the state of Sao Paulo, to record outpatient care, hospitalization, laboratory test results, in addition to prescribing and dispensing medicines.^a^

A total of 3,115 eligible patients were selected who had at least one consultation scheduled in 2007, 40 of which were excluded for being pregnant, showing inconsistencies regarding the information on sex and date of birth, being transferred from the service or having passed away before their first consultation. The analysis was therefore performed with 3,075 patients.

The outcome was missing a scheduled infectious diseases appointment. The absence rate for scheduled consultations was calculated for all the subjects, as were the percentage ratio between the number of absences and the total number of scheduled consultations during the period.

The independent variables for the study were: sex, age, unscheduled consultations, changing infectious diseases doctor, psychiatric consultations, psychological and social service care, hospitalization at the ICHCFMUSP and HAART. As regards the subjects undergoing HAART, the number of dispensed anti-retroviral drugs were compared among those who missed scheduled infectious diseases appointments and those who did not, in the 2007 period.

Information from SIGH regarding patient records, scheduling, hospitalizations and dispensed drugs were separately obtained and grouped in a single file formatted as a database in Microsoft Excel^®^ 2007, whose descriptive analysis of the variables was performed using this program, following the data’s quality being verified.^13^ ^,^ ^14^ ^,^ ^21^ The SPSS – Statistic software, version 21, was used to analyze the crude and multiple association among the variables. The variables for which p ≤ 0.20 were included in the analysis. p ≤ 0.05 was considered to be statistically significant.

This study was approved by the HCFMUSP Ethics Committee (Process 115/2009).

## RESULTS

We included 3,075 subjects, 70.0% of which were male. The mean age of the subjects was 44 years of age (SD = 9.3 years); 38.9% missed at least one scheduled appointment.

For the evaluated patients, there were 13,634 consultations scheduled in 2007 (mean of 4.4; SD = 1.9). The mean number of missed appointments was 1.6 per person (SD = 0.9; variation of 1 to 6). The general consultation absence rate was 13.9%, 5.2% of the patients missed almost 50.0% of their scheduled consultations (Table 1).

**Table 1:** People with HIV according to missed infectious diseases consultations. SEAP HIV/AIDS, 2007. (N = 1,195)

Consultations missed	People (n)	%	Consultations missed (n)	%	Rate of consultations missed (%)
1	715	23.3	715	37.7	21.2
2	321	10.4	642	33.9	36.0
3 to 6	159	5.2	539	28.4	49.8

There was an inverse association between age and absences observed (Figure). The mean age of the patients who missed consultations was 42.1 (SD = 9.8) years of age, which is lower than those who did not miss a consultation, which was 45.2 (SD = 9.4) years of age (p < 0.001).

**Figure:**
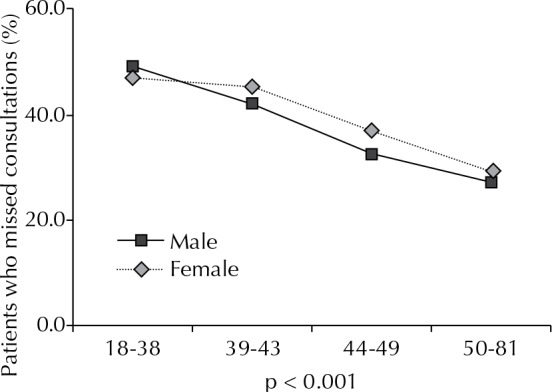
Proportion of people with HIV that missed infectious diseases consultations, according to sex and age.a,b SEAP HIV/AIDS, 2007.

^a^ 3,075 subjects included. Age in years grouped into quartiles.

^b^ Chi-square test for trend.

There was an inverse association between absences and HAART, there was also a direct association between absences and unscheduled infectious diseases consultations, care in social services, change of infectologist and hospitalization at ICHCFMUSP. There was no association with sex, psychiatric consultations or psychological care (Table 2).

**Table 2:** Description of people with HIV according to missed scheduled infectious diseases consultations. SEAP HIV/AIDS. 2007. (N = 3.075)

Characteristics		Missed		Did not miss		OR	p	95%CI

		n	%	n	%			
Sex	Male	826	38.3	1,331	61.7	1		
	Female	369	40.2	549	59.8	0.92	0.32	0.79;1.08
Unscheduled consultations	Yes	569	47.6	759	40.4	1		
	No	626	52.4	1,121	59.6	1.34	< 0.001	1.16;1.56
Change of doctor	Yes	265	22.2	354	18.8	1		
	No	930	77.8	1,526	81.2	1.23	0.03	1.03;1.47
Psychiatry	Yes	97	8.1	144	7.7	1		
	No	1,098	91.9	1,736	92.3	1.07	0.65	0.81;1.39
Psychology	Yes	112	9.4	155	8.2	1		
	No	1,083	90.6	1,725	91.8	1.15	0.28	0.89;1.49
Social service	Yes	368	30.8	448	23.8	1		
	No	827	69.2	1,432	76.2	1.42	< 0.001	1.21;1.67
ICHCFMUSP hospitalization	Yes	76	6.4	72	3.8	1		
	No	1,119	93.6	1,808	96.2	1.71	0.001	1.23;2.37
HAART	Yes	996	83.3	1,703	90.6	1		
	No	199	16.7	177	9.4	0.52	< 0.001	0.42;0.65

HAART: highly active antiretroviral therapy; ICHCFMUSP: *Instituto Central do Hospital das Clínicas da Faculdade de Medicina da Universidade de São Paulo* (Central Institute of the Clinical Hospital at the University of Sao Paulo’s Medical School)

During the multiple analysis, there was an observed association between absence and age, attending social services, receiving HAART, hospitalization at ICHCFMUSP and unscheduled consultation (Table 3).

**Table 3:** Multiple analysis of the factors associated with missed scheduled infectious diseases consultations. SEAP HIV/AIDS, 2007. (N = 3,075)

Characteristics	OR	P	95%CI
Age	0.97	< 0.001	0.96;0.97
Unscheduled consultations	0.81	0.008	0.70;0.95
Social service	0.72	< 0.001	0.61;0.85
ICHCFMUSP hospitalization	0.60	0.004	0.45;0.85
HAART	1.76	< 0.001	1.40;2.20
Change of doctor	1.18	0.084	0.98;1.41

HAART: highly active antiretroviral therapy; ICHCFMUSP: *Instituto Central do Hospital das Clínicas da Faculdade de Medicina da Universidade de São Paulo* (Central Institute of the Clinical Hospital at the University of Sao Paulo’s Medical School)

Among the analyzed subjects, 87.8% were undergoing HAART. Therefore, they dispensed their anti-retroviral drugs at the pharmacy service (on average, 10.1 [SD = 3.0]) per subject. Missed appointment was associated with the lowest number of antiretroviral drugs dispensed (Table 4).

**Table 4:** Frequency of dispensed antiretroviral drugs according to people with HIV who missed and did not miss their scheduled infectious diseases consultations SEAP HIV/AIDS, 2007. (N = 2,699)

	Frequency of dispensed antiretroviral drugs				p

	Mean	SD	Median	Min-Max	
Missed	8.7	3.35	10	1;15	< 0.001
Did not miss	10.8	2.51	12	1;17	

## DISCUSSION

Investigating missed scheduled consultations is a measure that is widely used in the literature to evaluate adhesion to treatment by PLHIV,^1^ ^,^ ^7^ ^,^ ^17^ the results of this study are similar to those from other publications.^16^ ^,^ ^20^ ^,^ ^23^

Despite the general rate of absences having been 13.9%, 5.2% of the cases had a rate of almost 50.0%. During other studies, this situation was associated to a higher risk of illness and death.^20^ ^,^ ^23^ Considering equity, according to the SUS, these subjects must be a particular target of care for the institution.

This study shows that the sex of the patients is not associated with missing consultations. This is a controversial association that is often described in the literature with varing results.^3^ ^,^ ^11^ ^,^ ^12^ ^,^ ^17^ ^,^ ^23^ Studies that focus on PLHIV highlight the association between younger individuals and missed appointments,^3^ ^,^ ^11^ ^,^ ^12^ ^,^ ^17^ which corroborates the findings of this study. However, there was a retrospective nationwide study conducted in China that showed missing consultations associated to individuals over the age of 60.^23^

The Brazilian National STD/AIDS Department from the Ministry of Health recommends that unscheduled consultations are available in services that assist PLHIV. The purpose of this recommendation is to ensure that those who missed their consultations, those with no medication or those showing some clinical complications still have access. Demand for unscheduled visits is expected to be low when the general organization of the service can meet most of the anticipated demands.^b^ The reduced demand for unscheduled consultations may have been caused by implemented routines designed to reduce access barriers; these are practiced in the HIV/AIDS Patient Care Extension Service such as rescheduling consultations by phone and making medical social benefit claims outside the consultation site.

Changing the doctor was not associated to absence, which is not the same as what was published in other studies.^3^ ^,^ ^8^ For PLHIV, changing the doctor may represent a loss of confidence in the treatment or professional, which results in missed consultations in addition to its impact on HAART adherence. Changing the doctor is a routine situation in services that are involved in medical education, namely with students and resident doctors.^8^

Subjects with the greatest adhesion have more access to the multidisciplinary team. This team must be made up of doctors from different specialties who are responsible for properly managing HIV, coinfections and comorbidities, as well as by nurses, social workers and psychologists. These individuals help PLHIV to overcome the various forms of suffering to which they are subjected and also play an important role in HAART adherence.^6,19,b^

There was an observed positive association between absence and care by social services, whose contribution to improve the quality of HIV/AIDS patient care has been highlighted by the National Department of STD/AIDS and Viral Hepatitis.^b^ During the period of study, the social services from the HIV/AIDS Patient Care Extension Service identified and helped PLHIV and their families to face up to social demands and make individual and group visits to patients with adherence difficulties. In addition, social service workers meet the unscheduled consultation demand, which makes access for patients with problems, including those who had missed the scheduled consultation, easier.

The association between HIV infection and depression has been observed in various studies.^4^ ^,^ ^15^ However, Bofill et al^1^ (2011) did not observe any such association between depression and missing medical consultations. In addition, despite drugs and alcohol having been associated to missed consultations,^12^ a study conducted in the HIV/AIDS Patient Care Extension Service did not identify this association.^10^ This study did not identify an association between missing consultations and attending psychological or psychiatric services, therefore other studies are needed to clarify this issue.

Previous studies have identified that HAART patients miss fewer consultations,^5^ ^,^ ^10^ ^,^ ^11^ ^,^ ^18^ ^,^ ^20^ which is a point that was noted in this study. Patients who miss consultations and are not undergoing HAART, despite this also having consequences, is probably considered less damaging than for those who are in treatment. This is explained by the fact that it is during the consultations that patients have routine access to anti-retroviral drug prescriptions. Furthermore, in 2007, HAART was offered to patients showing evidence of immunodepression, either due to a falling CD4 count or to illness, i.e., for patients in worse health conditions. Today, HAART should be offered to all PLHIV, regardless of their CD4 count. While offering HAART to these patients, health care professionals should make the patients aware of the possibility of committing to therapy, the purpose of which being to achieve the necessary adhesion so that, with HAART, the individual and collective benefits are achieved by those for which they were intended.

Missed appointments can represent a lost opportunity to treat and/or prevent illness.^20^ ^,^ ^23^ We observed an association between hospitalization at ICHCFMUSP and missed appointments. Only including hospitalizations at the ICHCFMUSP may be a limiting factor for this analysis.

It was possible to characterize the profile of PLHIV who missed their consultations using the SIGH. This system is in full operational use at the HCFMUSP and at other state health services. Data are available immediately after registration, and access to them is easy and fast.^21^

To obtain indicators related to PLHIV health care quality, secondary data were used, which is rarely used after completing the production and billing sheets. This type of study is particularly useful in the fight against the HIV/AIDS epidemic, in which the Brazilian National Department of STD, AIDS and Viral Hepatitis encourages treatment being adopted as a method of prevention. HAART, in addition to providing individual benefits, reduces the risk of HIV transmission from the moment the patient reaches an undetectable viral load.^9^

The most recent Brazilian Clinical Protocol and Therapeutic Guidelines for Managing HIV Infection in Adults,^c^ which was proposed by the Brazilian Department of STDs, AIDS and Viral Hepatitis from the Ministry of Health, recommends treatment for all persons with HIV infection, regardless of their CD4 count. Adhesion assessment becomes even more important in this situation. Low adhesion may compromise this strategy, causing the emergence of a large number of PLHIV with collating mutations that are resistant to anti-retroviral drugs (for which there would be few treatment options, many of them expensive) and the transmission of multi-drug resistant HIV.

Anti-retroviral drugs are made available throughout Brazil by the SUS. The Medication Logistics Control System (SICLOM) offers support to logistics and the distribution of these medicines. Once the necessary adjustments have been made, adherence evaluation can be done with secondary data generated by SICLOM. This is a simple and inexpensive form of monitoring adhesion, which can be used with all services that care for PLHIV undergoing HAART in Brazil. Brazil can and should use all its available resources rationally in the continued fight against the HIV/AIDS epidemic and continue on this bold and pioneering path.

## Footnotes

a Companhia de Processamento de Dados do Estado de São Paulo - PRODESP. Relatório de administração e de responsabilidade social 2010 [cited 13 Mar 2012]. Available from: http://www.prodesp.sp.gov.br/PRODESP_VALOR_ECONOMICO_2010.pdf

b Ministério da Saúde. Metas e compromissos assumidos pelos Estados membros na sessão especial da Assembleia Geral das Nações Unidas sobre HIV/aids UNGASS – HIV/aids. Resposta brasileira 2008-2009: relatório de progresso do país. Brasília (DF): Ministério da Saúde; 2010 [cited 2012 Mar 13]. Available from: http://www.aids.gov.br/publicacao/2011/orientacoes_da_onu_sobre_aids_ungass

c Ministério da Saúde, Secretaria de Vigilância em Saúde, Departamento de DST, Aids e Hepatites Virais. Protocolo clínico e diretrizes terapêuticas para manejo da infecção pelo HIV em adultos. Brasília (DF): Ministério da Saúde; 2013.
