# Supplementary material for: Characteristics of HIV patients who missed their scheduled appointments
Source: Rev Saude Publica. 2016 Jan 12;49:95. doi: 10.1590/S0034-8910.2015049005145 (PMC4716653; doi:10.1590/S0034-8910.2015049005145)
Supplement: Translation

# Translation: Características dos pacientes com HIV que faltaram a consultas agendadas

**Authors:** Delsa Nagata, Eliana Battaggia Gutierrez

**Affiliations:** I Programa de Pós-Graduação em Doenças Infecciosas e Parasitárias. Faculdade de Medicina. Universidade de São Paulo. São Paulo, SP, Brasil; II Divisão de Clínica de Moléstias Infecciosas e Parasitárias. Instituto Central do Hospital das Clínicas. Faculdade de Medicina. Universidade de São Paulo. São Paulo, SP, Brasil

**Keywords:** Síndrome de Imunodeficiência Adquirida, TARV, Adesão à Medicação, Continuidade da Assistência ao Paciente, Agendamento de Consultas, Sistemas de Informação em Saúde

## Abstract

**OBJETIVO:**

Analisar se características sociodemográficas e consultas e atendimentos em especialidades estão associados à falta de pessoas vivendo com HIV em consultas agendadas de infectologia.

**MÉTODOS:**

Neste estudo transversal e analítico foram incluídas 3.075 pessoas vivendo com HIV, com pelo menos uma consulta de infectologia agendada em unidade de saúde especializada em 2007. Foi utilizada base de dados secundários do Sistema de Informação e Gestão Hospitalar. A variável desfecho foi a falta em consulta agendada. As variáveis independentes foram sexo, idade, consultas e atendimentos em especialidades e disciplinas disponíveis, internações no Instituto Central do Hospital das Clínicas da Faculdade de Medicina da Universidade de São Paulo, tratamento antirretroviral e troca de médico infectologista. Realizada análise de associação bruta e múltipla entre as variáveis, com significância estatística se p ≤ 0,05.

**RESULTADOS:**

Mais de um terço (38,9%) dos pacientes faltaram em pelo menos uma consulta de infectologia; 70,0% dos pacientes eram do sexo masculino. A taxa de faltas foi de 13,9%, mas não foi observada associação entre sexo e falta. A idade foi inversamente associada à falta. Não estar em tratamento antirretroviral, comparecer à consulta de infectologia sem agendamento ou a atendimento de serviço social e ser internado no Instituto Central foram diretamente associados à falta.

**CONCLUSÕES:**

O Sistema de Informação e Gestão Hospitalar mostrou ser instrumento útil para a elaboração de indicadores relacionados à qualidade da atenção à saúde de pessoas vivendo com HIV. Outros sistemas de informação, frequentemente desenvolvidos para finalidades administrativas, também podem ser úteis para gestão local e regional e para avaliação da qualidade da atenção aos pacientes vivendo com HIV.

## INTRODUÇÃO

No contexto do acesso universal ao tratamento antirretroviral (TARV), um dos desafios para atingir maior efetividade e impacto da terapia de pessoas vivendo com HIV (PVHIV) é a manutenção de altas taxas de adesão ao TARV.^8^ ^,^ ^18^ O sucesso do TARV foi associado ao comparecimento às consultas médicas.^1^ ^,^ ^18^ Além disso, o número de faltas às consultas agendadas foi associado ao risco de óbito,^17^ ^,^ ^20^ ^,^ ^23^ e o total de dias perdidos entre a falta e a próxima consulta, com a ocorrência de novas doenças definidoras de aids.^20^

Sexo,^12^ idade,^3^ ^,^ ^11^ ^,^ ^12^ ^,^ ^17^ etnia,^3^ ^,^ ^12^ ^,^ ^17^ baixo CD4,^17^ uso de álcool e drogas,^17^ não estar em uso de medicamentos antirretrovirais,^11^ menor suporte social^3^ ^,^ ^17^ ou familiar^1^ e perda de seguridade social^17^ foram identificados como fatores associados à falta à consulta.

Do ponto de vista da gestão, a falta à consulta agendada é um problema para os serviços de saúde e para a sociedade e pode ser traduzido em custos.^2^ ^,^ ^22^ A forma como está organizado o serviço de saúde para o atendimento de PVHIV é aspecto importante relacionado à adesão ao TARV.^19^ Barreiras de acesso ao serviço e à informação, falta de regularidade no comparecimento às consultas agendadas e nas retiradas de medicamentos podem associar-se negativamente à adesão.^8^

O presente estudo teve por objetivo analisar se características sociodemográficas e consultas e atendimentos em especialidades estão associados à falta de pessoas vivendo com HIV em consultas agendadas de infectologia.

## MÉTODOS

Estudo transversal retrospectivo, analítico, desenvolvido no Serviço de Extensão ao Atendimento de Pacientes HIV/aids (SEAP HIV/aids) da Divisão de Clínica de Moléstias Infecciosas e Parasitárias do Instituto Central do Hospital das Clínicas da Faculdade de Medicina da Universidade de São Paulo (ICHCFMUSP), ambulatório e hospital dia especializado no atendimento de pessoas vivendo com HIV, em 2007. A unidade atendia aproximadamente 3.000 adultos com HIV por meio do Sistema Único de Saúde (SUS) no período. As consultas médicas eram agendadas por horário; também eram oferecidas consultas sem agendamento para atender a demanda espontânea.

As informações foram obtidas do Sistema de Informação e Gestão Hospitalar (SIGH), sistema administrativo hospitalar de produção e faturamento, desenvolvido pela Companhia de Processamento de Dados do Estado de São Paulo (PRODESP). O sistema é amplamente utilizado em serviços de saúde estaduais do SUS, no estado de São Paulo, para registro de atendimentos ambulatoriais, internações, resultados de exames laboratoriais, além da prescrição e dispensa de medicamentos.^a^

Foram selecionados 3.115 pacientes elegíveis com pelo menos uma consulta agendada em 2007, dos quais 40 foram excluídos devido à gestação, discordância nas informações sobre sexo e data de nascimento, transferência do serviço ou ao óbito antes da primeira consulta. Desta forma, a análise foi realizada com 3.075 pacientes.

O desfecho foi falta em consulta agendada de infectologia. A taxa de faltas em consultas agendadas de infectologia foi calculada para todos os sujeitos incluídos pela relação percentual entre o número de faltas e o total de consultas agendadas no período.

As variáveis independentes do estudo foram: sexo, idade, consultas sem agendamento, troca de médico infectologista, consultas de psiquiatria, atendimentos de psicologia e de serviço social, internação no ICHCFMUSP, TARV. Para os sujeitos em TARV, os números de retiradas de antirretrovirais foram comparados entre os que faltaram e os que compareceram a todas as consultas agendadas de infectologia em 2007.

As informações do SIGH sobre cadastro do paciente, agendamento, internações e retiradas de medicamentos foram obtidas separadamente e agrupadas em arquivo único em formato de banco de dados no programa Microsoft Excel^®^ 2007, cuja análise descritiva das variáveis foi realizada por esse programa, após verificação da qualidade dos dados.^13^ ^,^ ^14^ ^,^ ^21^ Para análise da associação bruta e múltipla entre as variáveis foi utilizado o programa SPSS – *Statistic*, versão 21. As variáveis para as quais p ≤ 0,20 foram incluídas na análise múltipla. Foi considerado p ≤ 0,05 como de significância estatística.

O estudo foi aprovado pelo Comitê de Ética do HCFMUSP (Parecer 115/2009).

## RESULTADOS

Foram incluídos 3.075 sujeitos, dos quais 70,0% eram do sexo masculino. A média de idade foi de 44 anos (DP = 9,3 anos); 38,9% faltaram em pelo menos uma consulta agendada.

Para os pacientes avaliados, foram agendadas 13.634 consultas em 2007 (média de 4,4; DP = 1,9). A média de faltas por pessoa foi de 1,6 (DP = 0,9; variação de 1 a 6). A taxa geral de faltas foi de 13,9%, mas 5,2% dos pacientes faltaram a quase 50,0% das consultas agendadas (Tabela 1).

**Tabela 1:** Pessoas com HIV segundo faltas em consultas de infectologia. SEAP HIV/aids, 2007. (N = 1.195)

Faltas	Pessoas (n)	%	Faltas (n)	%	Taxa de faltas (%)
1	715	23,3	715	37,7	21,2
2	321	10,4	642	33,9	36,0
3 a 6	159	5,2	539	28,4	49,8

Observamos associação inversa entre idade e faltas (Figura). A média de idade dos pacientes que faltaram foi 42,1 (DP = 9,8) anos, inferior à dos que não faltaram, 45,2 (DP = 9,4) anos (p < 0,001).

**Figura:** Proporção de pessoas com HIV que faltaram em consultas de infectologia, segundo sexo e idade.a,b SEAP HIV/aids, 2007.

^a^ Incluídos 3.075 sujeitos. Idade em anos agrupada em quartis.

^b^ Teste de Qui-quadrado de tendência.

Houve associação inversa entre falta e uso de TARV e direta entre falta e comparecimento a consultas de infectologia sem agendamento, atendimentos no serviço social, troca de médico, internação no ICHCFMUSP. Falta não apresentou associação com sexo, comparecimento a consultas de psiquiatria e atendimentos de psicologia (Tabela 2).

**Tabela 2:** Descrição de pessoas com HIV de acordo com faltas em consultas agendadas de infectologia. SEAP HIV/aids, 2007. (N = 3.075)

Características		Faltaram		Não faltaram		OR	p	IC95%

		n	%	n	%			
Sexo	Masculino	826	38,3	1.331	61,7	1		
	Feminino	369	40,2	549	59,8	0,92	0,32	0,79;1,08
Consultas sem agendamento	Sim	569	47,6	759	40,4	1		
	Não	626	52,4	1.121	59,6	1,34	< 0,001	1,16;1,56
Troca de médico	Sim	265	22,2	354	18,8	1		
	Não	930	77,8	1.526	81,2	1,23	0,03	1,03;1,47
Psiquiatria	Sim	97	8,1	144	7,7	1		
	Não	1.098	91,9	1.736	92,3	1,07	0,65	0,81;1,39
Psicologia	Sim	112	9,4	155	8,2	1		
	Não	1.083	90,6	1.725	91,8	1,15	0,28	0,89;1,49
Serviço social	Sim	368	30,8	448	23,8	1		
	Não	827	69,2	1.432	76,2	1,42	< 0,001	1,21;1,67
Internação ICHCFMUSP	Sim	76	6,4	72	3,8	1		
	Não	1.119	93,6	1.808	96,2	1,71	0,001	1,23;2,37
TARV	Sim	996	83,3	1.703	90,6	1		
	Não	199	16,7	177	9,4	0,52	< 0,001	0,42;0,65

TARV: tratamento antirretroviral; ICHCFMUSP: Instituto Central do Hospital das Clínicas da Faculdade de Medicina da Universidade de São Paulo

Na análise múltipla, observou-se associação entre falta e idade, comparecimento ao serviço social, receber TARV, internação no ICHCFMUSP e consulta sem agendamento (Tabela 3).

**Tabela 3:** Análise múltipla dos fatores associados às faltas em consulta agendada de infectologia. SEAP HIV/aids, 2007. (N = 3.075)

Características	OR	P	IC95%
Idade	0,97	< 0,001	0,96;0,97
Consultas sem agendamento	0,81	0,008	0,70;0,95
Serviço social	0,72	< 0,001	0,61;0,85
Internação ICHCFMUSP	0,60	0,004	0,45;0,85
TARV	1,76	< 0,001	1,40;2,20
Troca de médico	1,18	0,084	0,98;1,41

TARV: tratamento antirretroviral; ICHCFMUSP: Instituto Central do Hospital das Clínicas da Faculdade de Medicina da Universidade de São Paulo

Estavam em TARV 87,8% dos sujeitos. Portanto, retiravam antirretrovirais na farmácia do Serviço (em média, 10,1 [DP = 3,0]) por sujeito. Falta associou-se a menor número de retiradas de antirretrovirais (Tabela 4).

**Tabela 4:** Frequência de retiradas de antirretrovirais segundo pessoas com HIV que faltaram e que não faltaram em consultas agendadas de infectologia. SEAP HIV/aids, 2007. (N = 2.699)

	Frequência de retiradas de antirretrovirais				p

	Média	DP	Mediana	Mín - Máx	
Faltaram	8,7	3,35	10	1;15	< 0,001
Não faltaram	10,8	2,51	12	1;17	

## DISCUSSÃO

A falta em consulta agendada é uma medida amplamente utilizada na literatura para avaliar adesão de PVHIV ao tratamento,^1^ ^,^ ^7^ ^,^ ^17^ e os resultados deste estudo são semelhantes aos de outras publicações.^16^ ^,^ ^20^ ^,^ ^23^

Apesar de a taxa geral de faltas ter sido 13,9%, 5,2% da casuística teve taxa próxima a 50,0%. Esta situação foi associada, em outros estudos, ao maior risco de adoecimento e de morte.^20^ ^,^ ^23^ Considerando a equidade, de acordo com o SUS, esses sujeitos devem ser alvo de atenção especial por parte da instituição.

Neste estudo, sexo dos pacientes não apresentou associação com falta à consulta. Essa associação é controversa, descrita com frequência na literatura, com resultados variados.^3^ ^,^ ^11^ ^,^ ^12^ ^,^ ^17^ ^,^ ^23^ Estudos com PVHIV destacam a associação entre idade mais jovem e falta às consultas,^3^ ^,^ ^11^ ^,^ ^12^ ^,^ ^17^ corroborando o presente achado. Entretanto, estudo retrospectivo realizado na China, de abrangência nacional, mostrou a falta associada à idade superior a 60 anos.^23^

O Departamento Nacional de DST/aids do Ministério da Saúde do Brasil preconiza que consultas sem agendamento sejam disponibilizadas em serviços que assistem PVHIV. Isso para garantir o acesso daqueles que faltaram em consulta agendada, ficaram sem medicação ou apresentaram alguma intercorrência clínica. É esperada baixa procura por atendimentos sem agendamento quando a organização geral do serviço consegue responder à maioria das demandas previstas.^b^ A redução da procura pelas consultas sem agendamento pode ter sido causada pela implementação de rotinas para diminuir as barreiras de acesso, praticadas no SEAP HIV/aids, como solicitação de remarcação de consultas por telefone e solicitação de declaração médica para obtenção de benefícios sociais fora da consulta.

A troca de médico não se associou à falta, ao contrário de outros estudos publicados.^3^ ^,^ ^8^ A mudança de médico pode representar, para a PVHIV, perda de confiança no tratamento ou no profissional, resultando em falta à consulta, inclusive com impacto na adesão ao TARV. Troca de médico é situação rotineira em serviços que se destinam ao ensino médico, com alunos e residentes.^8^

Os sujeitos com maior adesão têm mais acesso à equipe multiprofissional. Esta deve ser composta por médicos de diversas especialidades, responsáveis pelo manejo adequado do HIV, das coinfecções e comorbidades, e por enfermeiros, assistentes sociais e psicólogos. Estes auxiliam as PVHIV a superar as várias formas de sofrimento a que estão submetidos e que têm importante papel na adesão ao TARV.^6^ ^,^ ^19^ ^,^ ^b^

Observamos associação positiva entre falta e atendimento pelo serviço social, cuja contribuição para melhorar a qualidade do cuidado ao paciente com HIV/aids tem sido salientada pelo Departamento Nacional de DST/aids e Hepatites Virais.^b^ No período do estudo, o serviço social do SEAP HIV/aids identificava e auxiliava as PVHIV e seus familiares no enfrentamento de demandas sociais e realizava atendimentos individuais e em grupo para pacientes com dificuldades de adesão. Além disso, os profissionais de serviço social atendiam a demanda sem agendamento, facilitando o acesso dos pacientes com problemas, inclusive os que haviam faltado à consulta agendada.

Associação entre infecção por HIV e depressão foi observada em diferentes estudos.^4^ ^,^ ^15^ Por outro lado, Bofill et al^1^ (2011) não observaram associação entre depressão e falta em consulta médica. Além disso, embora álcool e drogas tenham sido associados à falta em consulta,^12^ estudo realizado no SEAP HIV/aids não identificou essa associação.^10^ Embora neste estudo não tenha sido identificada associação entre falta e comparecimento a psicologia e a psiquiatria, outros estudos são necessários para esclarecer essa questão.

Estudos anteriores identificaram que pacientes em TARV faltavam menos em consultas,^5^ ^,^ ^10^ ^,^ ^11^ ^,^ ^18^ ^,^ ^20^ conforme observado neste estudo. A falta para os pacientes que não estão em TARV, embora também acarrete consequências, provavelmente é encarada de forma menos danosa do que para aqueles que estão em tratamento. Isso porque é na consulta que os pacientes têm acesso rotineiro à receita médica de antirretrovirais. Além disso, em 2007, o TARV era ofertado aos pacientes com evidências de imunodepressão, seja por queda de contagem de CD4 ou pela ocorrência de doença, i.e., para pacientes em piores condições de saúde. Hoje, o TARV deve ser ofertado a todas as PVHIV, independentemente da contagem de CD4. Ao ofertar TARV aos pacientes, os profissionais de saúde devem sensibilizá-los a firmar aliança terapêutica cuja finalidade é alcançar a adesão necessária para que, com o TARV, sejam alcançados os benefícios individuais e coletivos a que se destina.

A falta à consulta pode representar perda de oportunidade de tratamento e/ou prevenção de adoecimento.^20^ ^,^ ^23^ Observamos associação entre internação no ICHCFMUSP e falta às consultas. A inclusão apenas das internações realizadas no ICHCFMUSP pode ser um fator limitante para esta análise.

Foi possível caracterizar o perfil das PVHIV que faltaram às consultas por meio do SIGH. O sistema está em pleno funcionamento no HCFMUSP e em outros serviços de saúde estaduais. Os dados estão disponíveis logo após seu registro, e o acesso a eles é fácil e rápido.^21^

Para obter indicadores relacionados à qualidade da atenção à saúde de PVHIV, foram utilizados dados secundários, raramente utilizados após o preenchimento das planilhas de produção e faturamento. Este tipo de estudo é particularmente útil no enfrentamento da epidemia de HIV/Aids, em que o Departamento Nacional de DST, Aids e Hepatites Virais orienta a adoção do tratamento como prevenção. O TARV, além dos benefícios individuais, reduz o risco de transmissão de HIV a partir do momento em que o paciente atinge carga viral indetectável.^9^

O Protocolo Clínico e Diretrizes Terapêuticas para Manejo da Infecção pelo HIV em Adultos^c^ mais recente, proposto pelo Departamento de DST, Aids e Hepatites Virais do Ministério da Saúde, orienta tratar todas as pessoas com infecção por HIV, independentemente da contagem de CD4. A avaliação da adesão torna-se ainda mais importante nessa situação. A baixa adesão pode comprometer essa estratégia, causando a emergência de grande número de PVHIV com mutações conferidoras de resistência aos antirretrovirais (para os quais haveria poucas opções de tratamento, muitas delas caras) e da transmissão de HIV multirresistente.

Os antirretrovirais são disponibilizados em todo o Brasil pelo do SUS. O Sistema de Logística de Medicamentos (SICLOM) oferece apoio à logística e distribuição desses medicamentos. Feitas as devidas adaptações, a avaliação de adesão pode ser feita por dados secundários, gerados pelo SICLOM. É uma forma simples e barata de monitoramento de adesão, que pode ser utilizada em todos os serviços que atendem PVHIV em TARV no País. O Brasil pode e deve utilizar todos os recursos disponíveis racionalmente para continuar a enfrentar a epidemia de HIV/aids e prosseguir na trilha ousada e pioneira adotada.

## Footnotes

a Companhia de Processamento de Dados do Estado de São Paulo - PRODESP. Relatório de administração e de responsabilidade social 2010 [citado 13 mar 2012]. Disponível em: http://www.prodesp.sp.gov.br/PRODESP_VALOR_ECONOMICO_2010.pdf

b Ministério da Saúde. Metas e compromissos assumidos pelos Estados membros na sessão especial da Assembleia Geral das Nações Unidas sobre HIV/aids UNGASS – HIV/aids. Resposta brasileira 2008-2009: relatório de progresso do país. Brasília (DF): Ministério da Saúde; 2010 [citado 2012 mar 13]. Disponível em: http://www.aids.gov.br/publicacao/2011/orientacoes_da_onu_sobre_aids_ungass

c Ministério da Saúde, Secretaria de Vigilância em Saúde, Departamento de DST, Aids e Hepatites Virais. Protocolo clínico e diretrizes terapêuticas para manejo da infecção pelo HIV em adultos. Brasília (DF): Ministério da Saúde; 2013.

## DESTAQUES

Este estudo avaliou o perfil dos pacientes HIV-positivos que faltaram às consultas agendadas de infectologia em um ambulatório especializado em HIV, no qual são desenvolvidas, além da assistência, atividades de ensino e pesquisa.

Foram incluídos 3.075 pacientes, 70,0% do sexo masculino; 38,9% faltaram em pelo menos uma consulta de infectologia no ano avaliado; a taxa de faltas foi de 13,9%, mas 5,2% (159) dos sujeitos faltaram a quase 50,0% das consultas agendadas.

Não foi observada associação entre sexo e falta. Observamos associação inversa entre idade e faltas; a média de idade de pacientes que faltaram foi 42,1 (DP = 9,8) anos, inferior à dos que não faltaram, 45,2 (DP = 9,4) anos (p < 0,001). Não estar em tratamento antirretroviral, comparecer em consulta de infectologia sem agendamento e em atendimento de serviço social e ser internado no Instituto Central do HCFMUSP foram diretamente associados à falta.

A partir dos resultados, podemos sugerir que o Sistema Nacional de Saúde incorpore indicadores de risco para falta de adesão no cuidado a pessoas vivendo com HIV, tais como idade mais jovem, procura pelo Serviço Social e comparecimento fora da data do agendamento.

Rita de Cássia Barradas Barata

Editora Científica
